# Environmental aluminum oxide inducing neurodegeneration in human neurovascular unit with immunity

**DOI:** 10.1038/s41598-024-51206-4

**Published:** 2024-01-07

**Authors:** Yingqi Xue, Minh Tran, Yen N. Diep, Seonghun Shin, Jinkee Lee, Hansang Cho, You Jung Kang

**Affiliations:** 1 Institute of Quantum Biophysics, Sungkyunkwan University, Suwon, Republic of Korea; 2 Department of Biophysics, Sungkyunkwan University, Suwon, Republic of Korea; 3 Department of Intelligent Precision Healthcare Convergence, Sungkyunkwan University, Suwon, Republic of Korea; 4 School of Mechanical Engineering, Sungkyunkwan University, Suwon, Republic of Korea

**Keywords:** Immunology, Neuroscience, Pathogenesis, Risk factors, Engineering

## Abstract

Aluminum oxide nanoparticle (AlNP), a ubiquitous neurotoxin highly enriched in air pollution, is often produced as an inevitable byproduct in the manufacturing of industrial products such as cosmetics and metal materials. Meanwhile, ALNP has emerged as a significant public health concern due to its potential association with neurological diseases. However, the studies about the neurotoxic effects of AlNP are limited, partially due to the lack of physiologically relevant human neurovascular unit with innate immunity (hNVUI). Here, we employed our AlNP-treated hNVUI model to investigate the underlying mechanism of AlNP-driven neurodegeneration. First, we validated the penetration of AlNP across a blood–brain barrier (BBB) compartment and found AlNP-derived endothelial cellular senescence through the p16 and p53/p21 pathways. Our study showed that BBB-penetrating AlNP promoted reactive astrocytes, which produced a significant level of reactive oxygen species (ROS). The astrocytic neurotoxic factors caused neuronal damage, including the synaptic impairment, the accumulation of phosphoric-tau proteins, and even neuronal death. Our study suggests that AlNP could be a potential environmental risk factor of neurological disorders mediated by neuroinflammation.

## Introduction

Inhaled particulate matter (PM) has been nominated as a risk factor to human leading to acute or chronic health issues majorly in lung and heart^1,2^. Recently, other epidemiological studies have shown the strong correlation between PM and neurodegenerative disorders such as Alzheimer’s disease (AD) and Parkinson’s disease (PD)^3,4^. Airborne aluminum oxide nanoparticle (AlNP), one of the components forming agglomerates with PM in air pollutants is primarily emitted by industrial cosmetic or metal-produced factories^5^. The concentrations of AlNPs are in the range of 1.1–7.3 mg/m^3^ per day in these industrial areas^6^. Several studies suggested that AlNPs could be the critical element as it could target the central nervous system (CNS) and potentially contributing to neurocytotoxicity^7–9^. Current studies showed the possibility that the inhaled AlNPs can migrate to the brain through the olfactory pathway or the circulation pathway^8,10^. In addition, AlNPs showed inherent toxicity, which would lead to intellectual or memory harmful influence on human health risks^11^. Mirshafa et al. reported that blood–brain barrier (BBB)-penetrating ALNPs caused the oxidative stress and mitochondrial damage in rat brain tissues, alarming the potential risk of AlNPs in the human brain^7^. In addition, among the immune system, glia activation, including astrogliosis and microgliosis, was observed in AlNP-exposed rat brains, which further led to significant neuronal damage^12^. However, the underlying mechanisms of AlNPs penetration to the brain and consequences of BBB penetration leading to neuroinflammation and neuronal damage are not clarified yet due to the limited model system recapitulating human neurovascular unit (hNVU) with innate immunity (hNVUI) in the human brain. In this regard, it is crucial to investigate the potential penetration activity of AlNPs to brain area and their contributions to neuropathology.

Given the fact that the deposition of nanoparticles (10–100 nm) was found in the alveolar area dominantly^13,14^, it appeared that the BBB-penetration route would be more responsible for the entrance of AlNPs into the brain. In addition, we previously validated that PMs disrupted BBB tightness and penetrated the brain leading to neuroinflammation and neuronal damage^15^. Here, we hypothesized that AlNPs would enter the brain passing through the BBB route and initiate neuroinflammation, which would further increase neuronal damage at the end. To test our hypothesis, we first developed AlNP-treated hNVUI model in the microfluidics consisting of two compartments: a blood vessel compartment where endothelial cells (ECs) were forming BBB exposed to AlNPs and a brain compartment where neurons and astrocytes were receiving soluble factors from the blood compartment. We first validated the impaired adjacent contacts of BBB resulting from AlNP-induced endothelial senescence through p21 and p16 pathways, which facilitated the transport of AlNPs to the brain side. We then observed that BBB-penetrating AlNPs promoted the reactivity of astrocytes releasing excessive reactive oxygen species (ROS). Finally, we demonstrated the accumulation of phosphoric-tau (pTau) was induced by prolonged AlNPs-activated astrocytes, triggering the exacerbation of synapse damage and neurodegeneration ultimately. Taken together, these findings provide valuable insight for further investigations and the development of therapeutic drugs targeting AlNP-induced neurodegenerative diseases.

## Results

### Development of hNVUI model

**Figure 1:**
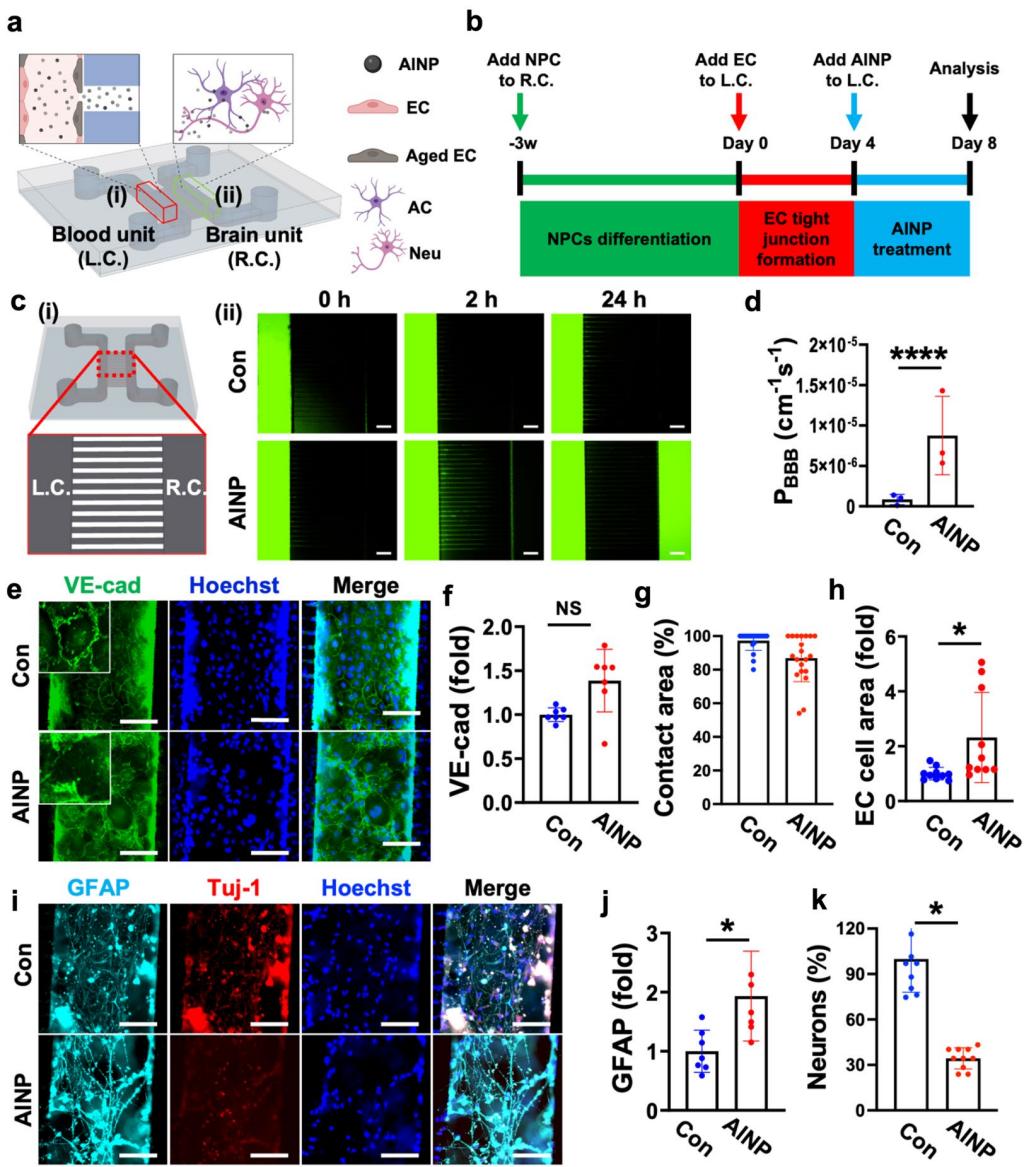
Implementation of human neurovascular unit with immunity (hNVUI) to assess neurotoxic effects of Aluminum oxide nanoparticles (AlNPs). (**a**) Schematic diagram of hNVUI platform to study: (i) AlNP penetration across blood–brain barrier (BBB) and translocation to brain area and (ii) AlNP-driven neuroinflammation leading to neurodegeneration. L.C. represents a left-side chamber; R.C. represents a right-side chamber. (**b**) Experimental timeline for the AlNP-treated model preparation. (**c**) Effects of AlNPs on the diffusion of FITC-dextran passing through BBB. FITC-dextran (10 μM, M.W. 40 kDa, green) was added to the lumen side of L.C. and the diffusion of FITC-dextran toward R.C. was monitored for 24 h. (**d**) Assessment of permeability coefficient of 40 kDa FITC-dextran with or without AlNPs for BBB formed in L.C. (**e**) Fluorescent images of endothelial cells (ECs) after exposed to control media (Con) and 1 ng/mL of AlNPs in L.C. side, which were stained with VE-cad and Hoechst indicating EC’s tight-junctions and nucleus, respectively. (**f**–**h**) Quantitative results of (**f**) VE-cad expression, (**g**) EC contact area, and (**h**) EC size were measured from (**e**) and represented as bar graphs. (**i**) Fluorescent images of reactive astrocytes and neurons in R.C. side marked by GFAP and Tuj-1. (**j**–**k**) Quantitative results of (**j**) GFAP, and (**k**) Tuj-1 were measured from (**i**) represented as bar graphs. Scale bars, (**c**) 500 μm and (**e**, **i**) 100 μm. Inset scale bars, 20 μm. All data are presented as mean ± SD measured by two-tailed unpaired Student’s t-test. *, *p* < 0.05 and ****, *p* < 0.0001.

To investigate the effects of AlNPs on the BBB permeability and their underlying mechanisms leading to neurodegeneration, we developed the AlNP-treated hNVUI model for the first time. This microfluidic-based model was comprised of two tube-shaped compartments, one for the growth of ECs forming BBB (Blood unit) in the left-side compartment (L.C.) and another for the growth astrocytes/neurons (Brain unit) in the right-side compartment (R.C.) (Fig. 1a). We designed to connect Blood and Brain units through multiple microchannels allowing the transport of AlNPs from the blood to the brain area and reciprocal exchange of soluble factors between two compartments. Experimental timeline for hNVUI model preparation was summarized in Fig. 1b. Briefly speaking, we cultured human ReNcell VM human neural progenitor cells in the Brain unit (R.C.) precoated with poly D-lysine (PDL) and Matrigel compartment, which were differentiated into neurons and astrocytes under the serum starvation for around 3 weeks. Upon the completion of differentiation, h3MEC/D3 ECs were loaded into the Blood unit (L.C.) precoated with Collagen IV and cultured in serum-free media for 4 days aiming to accelerate the formation of tight BBB. Upon the completion of hNVUI development, we added 1 ng/mL of AlNPs to the ECs (AlNP) for 4 days. It should be noted that the concentration of AlNPs found in non-industrial areas is around 5 × 10^–4^ ng/mL while that in the industrial area is exceeding ~ 1 ng/mL according to ACGIH^6^. Thus, we selected 1 ng/mL as the concentration of AlNPs for this study, which has reached the pathological range^6,16,17^. For the control counterpart, we added the basal media without AlNPs (Con).

Prior to testing potential risks of AlNPs on the BBB, we validated the formation of tight-junctions properly in the BBB side of our model by estimation of an apparent permeability coefficient of 40 kDa fluorescein isothiocyanate (FITC)–dextran (Pe = 8.5 × 10^−7^ cm/s)^15,18,19^. To explore the influence of AlNPs on the BBB, we added the FITC-dextran along with or without AlNPs to the L.C. side and monitored any penetration of the fluorescent molecules to the R.C. side (Fig. 1c-i). As shown in Fig. 1c-ii, the treatment of AlNPs facilitated the dye permeated across the lumen of BBB entering the brain unit. Our data showed that the treatment of AlNPs increased the permeability coefficient of FITC-dextran significantly (Pe = 8.8 × 10^−6^ cm/s) (Fig. 1d). To investigate the mechanism disrupting the BBB tightness, we assessed the expression levels of vascular endothelial cadherin (VE-cad) and Zonula Occludens-1 (ZO-1), specific markers for the adherent junction (Fig. 1e,f) and the tight junction (Supplementary Fig. 1) to determine the integrity of BBB. Our data showed that the expression of VE-cad in AlNP-exposed ECs was not significantly changed compared to control counterpart (Fig. 1f). Similarly, no significant change was observed in the expression of ZO-1 in AlNP-treated ECs compared to controls (Supplementary Fig. 1). Even if there was no change in the VE-cad and ZO-1 expression level, we found that the expression level of VE-cad on the cell–cell contact area was significantly decreased (Fig. 1g). Our data indicated that AlNPs may increase permeability through alternating the distribution rather than changing the expression of adhesion proteins^20^. Interestingly, the morphology of cells exposed to AlNP was shown to be enlarged, swollen and multinucleated, indicating a potential occurrence of endothelial senescence, which would promote BBB permeability. To quantify the endothelial cell area, ECs treated with or without AlNPs were randomly captured and their surface area was measured t. We found that the AlNP-treated ECs enlarged 2.32-fold than the control (Fig. 1h), supporting our conjecture that these AlNP-treated cells were undergoing senescent procedure. We next estimated the concentration of AlNPs penetrating BBB by Nanoparticle Tracking Analysis (NTA) and found that about 14.6% of AlNPs were penetrating BBB per a day (Supplementary Fig. 2). These results demonstrated that AlNPs were capable of penetration into the brain side, which would damage the brain side.

To investigate whether BBB-penetrating AlNPs could affect the brain, we checked expression levels of glial fibrillary acidic protein (GFAP) and β Tubulin 3/Tuj1 (Tuj-1) in the brain side of hNVUI models, markers indicating the reactivity of astrocytes and neural population (Fig. 1i). Our data revealed the significant increase in the reactivity of single astrocytes (1.93-fold) in AlNP-treated hNVUI models (Fig. 1j), which would promote neuroinflammation increasing the neuronal damage. To further confirm the astrocyte activation induced by the uptake of AlNPs, we monitored the activity of microtubule-associated protein light-chain 3 (LC3)-associated phagocytosis by assessing the any upregulation of LC3b, the active form of LC3 (Supplementary Fig. 3)^21^. Our data exhibited that AlNP-treated astrocytes expressed a higher level of LC3b compared to controls, indicating the AlNPs can induce LC3-mediated phagocytosis, which would activate astrocytes in advance. Correspondingly, we observed a significant reduction in the population of neurons (Fig. 1k) along with the promoted formation of cleaved caspase-3 (C-Cas-3), a marker representing the apoptotic cell death (Supplementary Fig. 4)^22^. It should be noted that the entire viability (Supplementary Fig. 5a) and the cell population (Supplementary Fig. 5b) in the brain side were not changed significantly as the number of astrocytes (ALDH1A1^+^ cells) was increased (1.94-fold) (Supplementary Fig. 6), which would compensate the reduction in neural population. Overall, our study revealed that BBB-penetrating AlNPs could enhance the astrocytic reactivity and neurodegeneration.

### Induction of senescent ECs under AlNP-enriched environments

**Figure 2:**
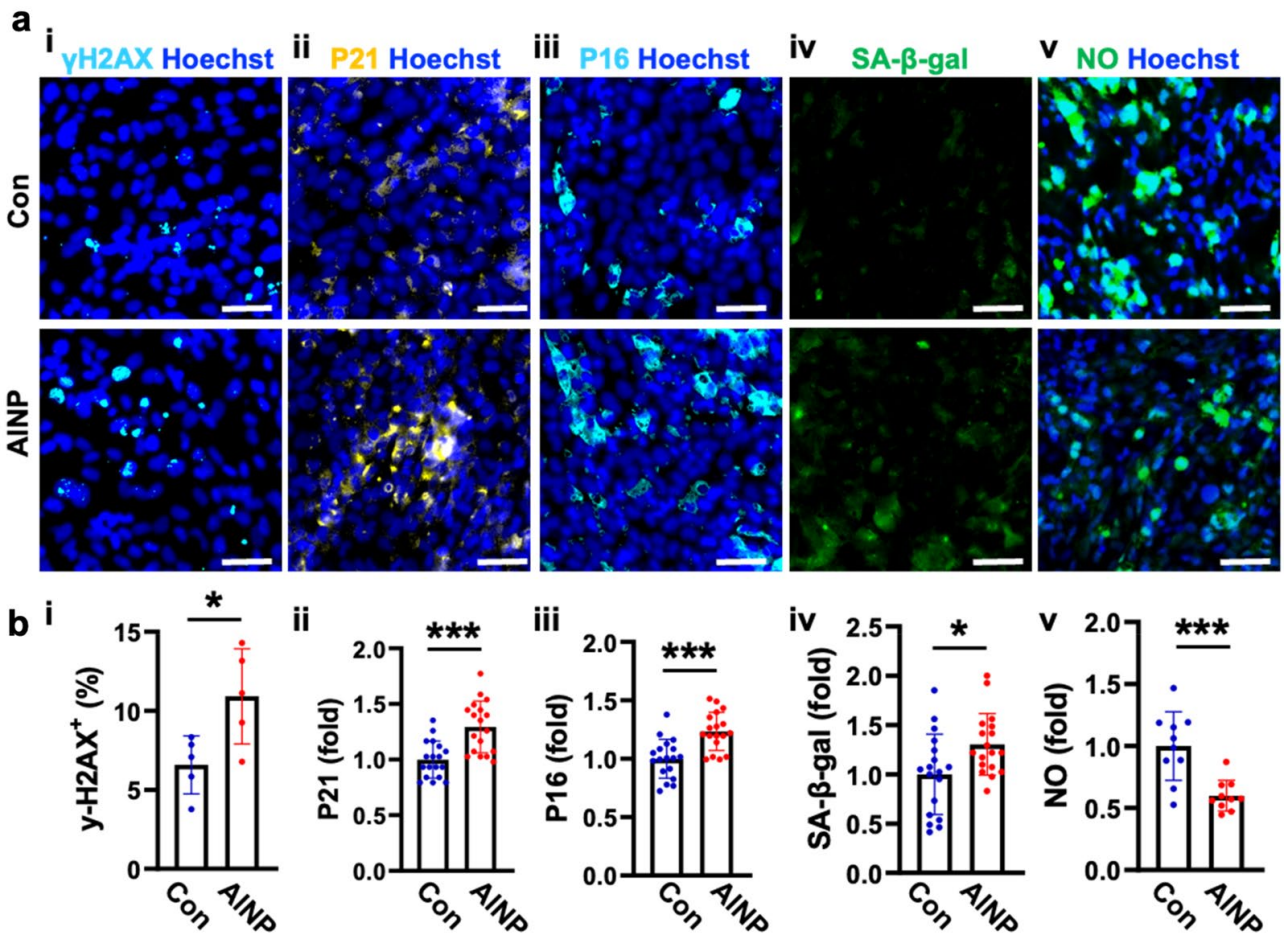
Cerebral endothelial senescence caused by AlNP. (**a**) Immunostaining images validating endothelial senescence. ECs were treated with control medium (Con) or AlNPs at 1 ng/mL for 4 days showing (i) DNA damage, (ii, iii) cell-cycle arrest, (iv) senescence, and (v) functional impairment, respectively. (**b**) Quantitative analyses in bar graphs. Scale bars, 50 μm. All data are presented as mean ± SD measured by two-tailed unpaired Student’s t-test. *, *p* < 0.05 and ***, *p* < 0.001.

Next, our study investigated the underlying mechanisms contributing to the AlNP-driven BBB impairment. Previous study suggested that metal-based nanoparticles (NPs) elevated BBB permeability, facilitating their penetration through BBB^23^. The exposure to fine dusts involving metal components has been related to the downregulation of nitric oxide synthase (eNOS) in EC, which contributed to senescence-related EC dysfunction^24^. Correspondingly, we observed the increased number of enlarged ECs presumably undergoing the senescence in the Blood compartment by AlNPs. In this regard, we here hypothesized that AlNPs could be the major component in PMs attributing the induction of EC senescence and leading to the leaky BBB in hNVUI models. To test the hypothesis, we prepared the simple BBB model by seeding ECs in 96 well plate and treated the model with either 1 ng/mL of AlNPs or culture media as a control for 4 days. We firstly checked any induction of DNA damage in AlNP-treated BBB by immunostaining against gamma-H2AX (γH2AX) (Fig. 2a-i). Approximately 10.9% of AlNP-treated ECs were γH2AX foci-positive, which was significantly increased compared to control (6.6%), indicating that AlNP treatment resulted in the induction of DNA damage in ECs (Fig. 2b-i). We further analyzed the expression levels of p21 (Fig. 2a-ii) and p16 (Fig. 2a-iii), which were recognized as two major signaling pathways contributing to cell cycle arrest after DNA damage leading to cellular senescence^25^. Our immunofluorescence data showed that the treatment of AlNPs resulted in the increase of both p21 (1.29-fold) (Fig. 2b-ii) and p16 (1.23-fold) (Fig. 2b-iii), validating the ECs were undergoing senescence. To validate the induction of senescence by AlNP treatment, we performed immunostaining against a gold standard senescence marker, beta-galactosidase (SA-β-gal) (Fig. 2a-iv). The increase in the number of SA-β-gal-positive cells was observed in the BBB models treated with AlNPs (Fig. 2b-iv), further supporting our hypothesis that the exposure to AlNP-enriched environment can induce cellular aging and cause cell senescence. Recent studies showed that cellular senescence was related to the arterial dysfunction decreasing the production of nitric oxide (NO), the basic element playing pivotal roles on immune modulation^26^. In this regard, we assessed the level of NO to check any impairment in BBB function induced by AlNP-driven senescence (Fig. 2a-v,b-v). As depicted in Fig. 2b-v, the level of NO was decreased to 0.6 times following the AlNP treatment, which strongly indicated the potential impairment of immune homeostasis in ECs attributed to AlNP-driven senescence. Taken together, data above represented that AlNP exposure can cause the DNA damage in ECs, which activated the expression of p16 and p21, subsequently resulting the hyperactivation of SA-β-gal contributing to the cell senescence as well as the impairment of BBB functions as the reduced NO production.

### Activation of reactive astrocytes in response to AlNP

**Figure 3:**
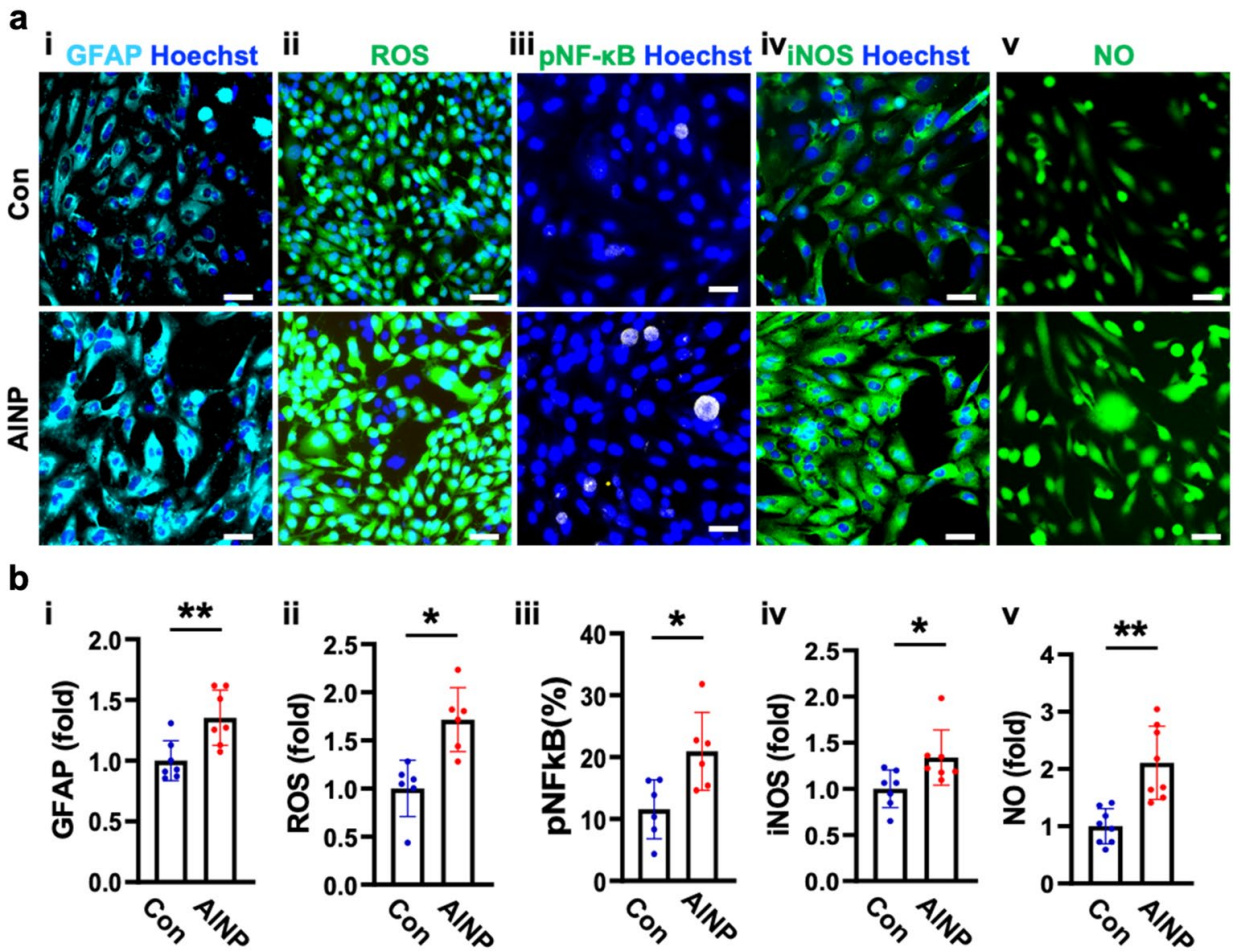
Neurotoxic astrocytic reactivity induced by AlNP. (**a**) Astrocytes were treated with control or AlNPs at 1 ng/mL for 2 days showing (i) astrocytic activation, (ii–v) ROS-mediated NF-κB pathway. (**b**) Quantitative analyses in bar graphs. Scale bars, 50 μm. All data are presented as mean ± SD measured by two-tailed unpaired Student’s t-test. *, *p* < 0.05 and **, *p* < 0.01.

We investigated the underlying mechanisms regarding the induction of reactive astrocytes in AlNP-treated hNVUI models. Due to the capability of AlNPs to reach to the brain compartment as shown in Fig. 1d and Supplementary Fig. 2, we hypothesized that BBB-penetrating AlNPs would directly affect astrogliosis. To test our hypothesis, we prepared the model with single-cultured astrocytes treated with or without 1 ng/mL of AlNPs for 2 days and monitored the phagocytosis activity as well as reactivity of astrocytes. As we observed in Supplementary Fig. 3, AlNP-treated astrocytes showed the increased phagocytic activity (Supplementary Fig. 7a) and the promoted level of LC3b (Supplementary Fig. 7b). Accordingly, we found the increased expression level of GFAP, a marker for the reactive astrocyte indicating the activation of early immune response toward pathogens (Fig. 3a-i,b-i). Data showed that the GFAP level was increased in astrocytes stimulated by AlNPs (1.4-fold) compared to controls, suggesting the induction of astrogliosis by AlNPs (Fig. 3b-i). We next checked any accumulation of reactive oxygen species (ROS) (Fig. 3a-ii,b-ii), one of the major underlying mechanisms increasing the reactivity of astrocytes^27^. Our data validated the significant accumulation of ROS in AlNP-treated astrocytes compared to controls (1.7-fold) (Fig. 3b-ii), indicating that AlNPs promoted the elevation of ROS in astrocytes that would further drive the induction of reactive astrocytes. To further demonstrate the inflammatory response of the reactive astrocytes driven by AlNP treatment, we checked the level of phospho-NF-κB p65 (pNF-κB), a gold standard marker for the activation of proinflammation in astrocytes^28^ (Fig. 3a-iii,b-iii). In accordance with previous data, we found the notable pNF-κB activation in AlNP-stimulated astrocytes compared to controls. Since ROS-mediated NF-κB pathway was known to activate inducible nitric oxide synthase (iNOS)^29^, the proinflammatory response producing neurotoxic NO^15^, we monitored the levels of iNOS (Fig. 3a-iv,b-iv) and NO (Fig. 3a-v,b-v) in the astrocytes. Our data validated that AlNP-exposed astrocytes expressed higher levels of iNOS and NO, which were 1.3-fold (Fig. 3b-iv) and 2.1-fold (Fig. 3b-v) higher than those without AlNP stimulation. These data indicated that the neuroinflammatory response of astrocytes was triggered under the AlNP existing environment. To further specify the type of astrocytes induced by AlNPs, we investigated the population of A1-like reactive astrocytes (complement component 3 (C3)-positive astrocytes), the proinflammatory phenotype presenting in most major neurodegenerative diseases (Supplementary Fig. 8)^30^. A few C3-positive astrocytes were observed without the treatment of AlNPs while a significant percentage of astrocytes showed C3-positive with the exposure of AlNPs, indicating that the induction of A1-like astrocytes by AlNP treatment. Taken together, our results validated that BBB-penetrating AlNPs could trigger the activation of A1-like astrocytes through ROS-mediated NF-κB pathway, serving proinflammatory roles in the brain.

### AlNP-exposed EC conditioned media slightly induced astrogliosis

**Figure 4:**
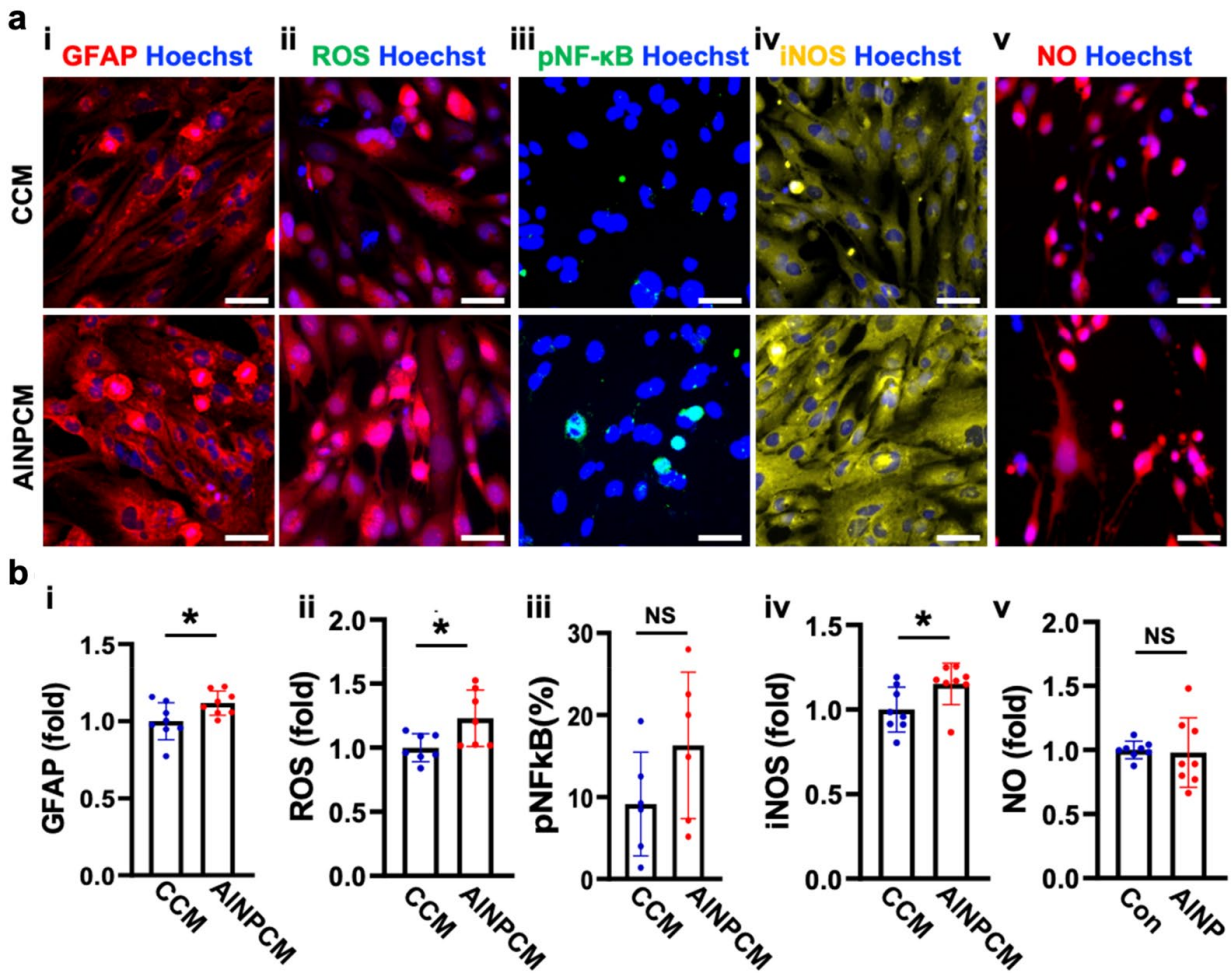
Astrocytic reactivity augmented by ALNP-treated endothelial cells. (**a**) Astrocytes were treated with conditioned media from non-treated ECs (CCM) or conditioned media from AlNP-treated ECs (ALNPCM) for 2 days showing (i) reactivation of AC, (ii–v) neuroinflammation led by the ROS-mediated NF-κB pathway. (**b**) Quantitative analyses in bar graphs. Scale bars, 50 μm. All data are presented as mean ± SD measured by two-tailed unpaired Student’s t-test. *, *p* < 0.05, and ns, no significance.

ECs forming the BBB layer are known to face pathogens or pollutants and release proinflammatory cytokines initiating defense mechanisms in the normal brain^31^. In the neurological disorders including vascular dementia, AD, and PD cases, damaged ECs forming the leaky BBB were frequently observed, which further released proinflammatory cytokines and toxic factors facilitating neurodegeneration^32,33^. In this regard, we tested whether soluble factors derived from AlNP-exposed ECs could promote any inflammation, particularly focusing on astrogliosis. To this end, we collected the conditioned media from AlNP-treated ECs (AlNPCM) and treated single-cultured astrocytes for 2 days. For the control counterpart, we treated the astrocytes with the conditioned media of non-treated ECs (CCM) for 2 days. Afterward, we monitored changes in astrogliosis markers as shown in the previous section as GFAP, ROS, pNF-κB, iNOS, and NO (Fig. 4). The quantification results indicated that GFAP, ROS and iNOS were slightly increased as 1.1-fold, 1.2-fold, and 1.2-fold respectively, while NO and pNF-κB did not show statistical significance. Accordingly, the endothelial-induced reactive astrocytes, expressing high level of Decorin, were slightly increased by AlNPCM (Supplementary Fig. 9). Overall, we concluded that AlNP-stimulated ECs had lesser effect on the astrogliosis contributing to neuroinflammation.

### Neurotoxic proinflammation driven by reactive astrocytes in AlNP-treated brains

**Figure 5:**
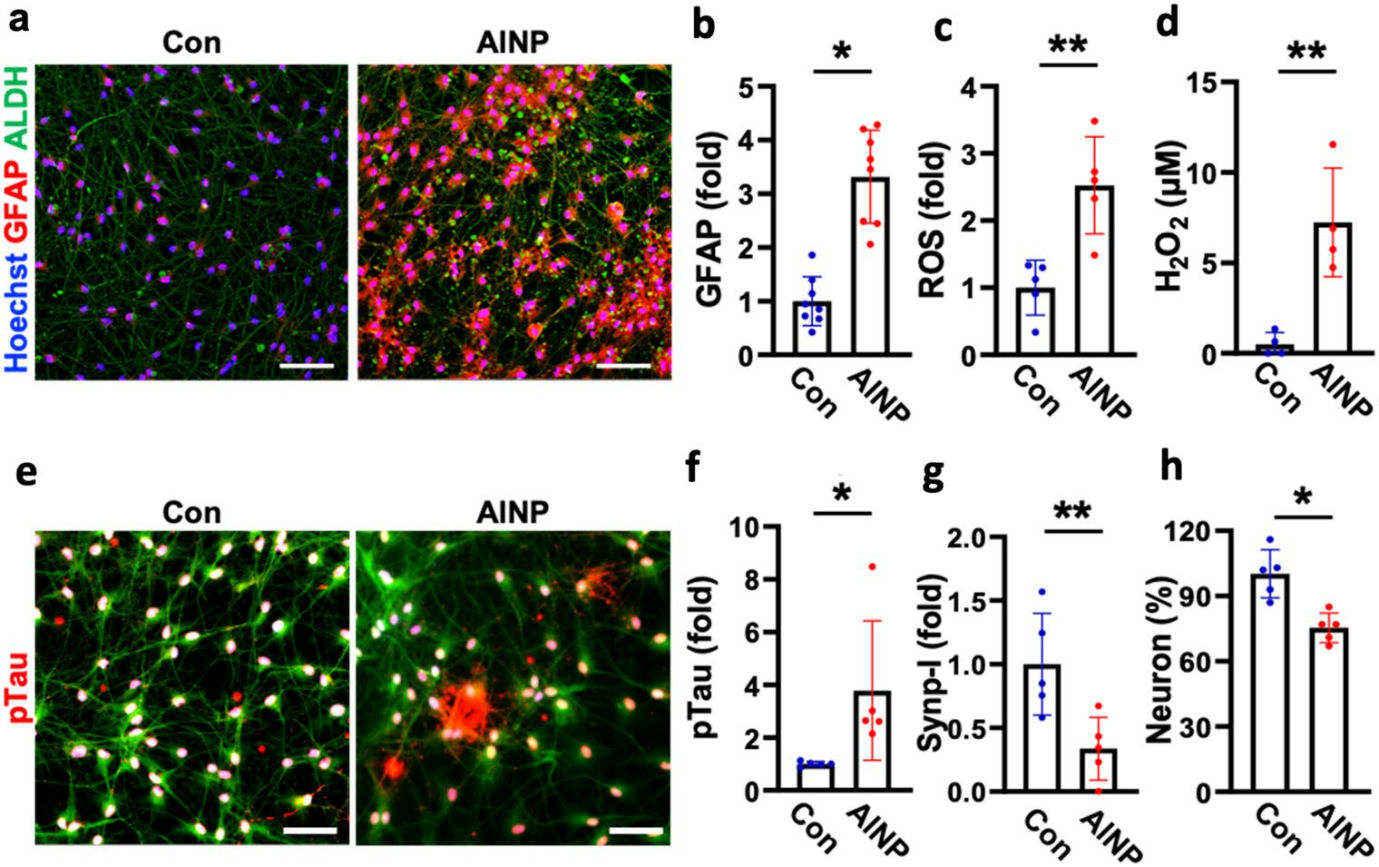
Neurodegeneration caused by AlNP-reactive astrocytes. (**a**) Immunostaining images of co-cultured neurons and astrocytes showing the discernable reactivity of astrocytes with AlNP compared to control. (**b**–**d**) Quantification analysis confirmed the activation of reactive astrocytes by long-term treatment of AlNP as (**b**) elevation of GFAP expression, (**c**) accumulation of ROS, and (**d**) production of H_2_O_2_. (**e**–**h**) Verification of neurodegeneration evidenced by accumulated (**f**) pTau, (**g**) reduced synapsin, and (**h**) diminished neuronal populations under chronic exposure of AlNPs. Scale bars, 50 μm. All data are presented as mean ± SD measured by two-tailed unpaired Student’s t-test. *, *p* < 0.05 and **, *p* < 0.01.

We next investigated the effect of chronic AlNP exposure on the neuronal damage by using the co-cultured model of neurons and astrocytes we described previously^15^. It should be noted that we selected the treatment condition for the chronic model as 10 pg/mL of AlNPs for 4 weeks corresponding to the concentration reaching to the brain by passing through BBB (Supplementary Fig. 2). Also, this condition was in the range of AlNP concentrations not affecting cell viability of ECs and astrocytes but promoting neuroinflammation based on our preliminary studies (Supplementary Figs. 11 and 12). Considering previous findings that short-term AlNP treatment activated astrocytes and induced neuronal death, we assumed that chronic AlNP treatment would induce reactive astrocytes and further promote neurodegeneration as well. We firstly checked the any proinflammatory response by astrocytes under the chronic AlNP exposure (Fig. 5a–c). The immunostaining results showed GFAP and ROS in astrocytes were significantly increased as 3.3-fold (Fig. 5b) and 2.5-fold (Fig. 5c) respectively, indicating that astrocytes retained hyperactive status in response to the continuous AlNP-enriched environment. We previously showed that the severe activation of astrocytes could generate excessive hydrogen peroxide (H_2_O_2_), causing oxidative stress contributing to neuronal death and taupathy^33^. Thus, we collected the conditioned medium from co-cultured models with or without AlNP long-term treatment and assessed the production level of H_2_O_2_ by the reactive astrocytes (Fig. 5d). The results showed significant increases in the production of H_2_O_2_ (7.2 μM) in AlNP-treated chronic model compared to controls, corresponding to the concentrations promoting tauopathy and exacerbating neurological disorders^15,33,34^.

Finally, we explored any toxic effects of chronic AlNP exposure on the neuronal cells. Interestingly, we observed the hyper deposition of pTau in the chronic AlNP models (Fig. 5e,f, 3.8-fold compared to controls), a critical component promoting neuronal damage in neurological disorders, such as AD, PD, and other types of dementia^35^. As the synapses enable the chemical or electrical signals transmitted between neurons, the impairment of synapses can cause neuronal death or even trigger neurological diseases^36^. We found that chronic exposure of AlNP caused the notable loss of synapses between neurons (Synp-1) as 33.7% compared to controls (Fig. 5g). Corresponding to induction of tauopathy and loss of synapses by the chronic AlNP condition, the population of NeuN-positive cells significantly decreased to 75.4% (Fig. 5h). We presumed that H_2_O_2_ generated from reactive astrocytes under chronic AlNP condition would trigger tauopathy, synaptic loss, and neurodegeneration at the end. Further studies will be required to confirm H_2_O_2_-driven tauopathy, which would proceed neurodegeneration.

## Discussion

Previous independent studies have shown that the airborne AlNP, one of the risk factors existing in PM, can penetrate BBB and invade the brain, triggering the glia activation followed by oxidative stress^7,12^. However, it is still challenging for current animal models to thoroughly elucidate the underlying mechanisms of BBB leakage contributing to astrogliosis and neurodegeneration under the exposure of AlNPs. Recently, emerging microfluidic techniques for cell culture have been allowed to develop human mini-brains recapitulating pathophysiological features found in neurological disorders^37–39^. Here, we developed an hNVUI model in the microfluidics consisting of Brain and Blood vessel compartments that can clearly recapitulate the whole brain-invaded route of AlNPs. While our microfluidic hNVUI model effectively recapitulated brain pathophysiology, it still exhibited certain limitations, including the lack of pericytes which would serve an important role in regulating the gene expression in ECs affecting BBB functions. Moreover, the microchannels that separated ECs from astrocytes did not fully replicate the natural configuration such as the close contact of astrocytic end feet to the EC. Additionally, the limited survival duration of ECs in our hNVUI model posed a significant challenge for conducting the long-term study. Therefore, we aim to develop more physiologically relevant models in the future that would offer a faithful platform for mechanisms study and drug screening.

This study also confirmed that the BBB-penetrating AlNPs promoted reactive astrocytes while soluble factors from AlNP-treated BBB minorly affected them. We recently reported that the induction of reactive astrocytes in response to pathogens or insoluble deposits in the brain and further contributed to neurodegeneration^33,34^. In accordance with these studies, we discovered that astrocytes retained the reactive phenotype expressing the high level of GFAP in response to AlNP treatment (Fig. 3a-i). Among the reactive population, we clarified that AlNPs promoted the population for A1-like reactive astrocytes up to 22% (Supplementary Fig. 8); therefore, it would be valuable to consider other types of astrocytes offering neuroprotection, such as A2-like astrocytes in the future study. In addition, our study indicated that only a few astrocytes were activated after the treatment with soluble factors from AlNPCM, with slight increase in GFAP (Fig. 4a-i). We observed that only 7% of astrocytes were influenced by endothelial cells (Supplementary Fig. 9). Moreover, there was no significant change in the pNF-κB (Fig. 4a-iii) of single-cultured astrocytes treated with AlNPCM compared to controls. Correspondingly, ALNPCM-treated astrocytes did not produce significant levels of proinflammatory cytokines (Supplementary Fig. 10). Therefore, it appears that the primary cause of astrocyte reactivation would be the penetrated AlNPs, rather than the influence of the inflammatory cytokines present in AlNPCM.

Finally, our data elucidated that AlNPs could initiate neurodegeneration mediated by reactive astrocytes. Our previous study showed that severe astrogliosis could increase the production of H_2_O_2,_ which further promoted tauopathy and initiated neurodegeneration^33^. Similarly, we found that co-culture of reactive astrocytes driven by AlNPs and neurons increased the H_2_O_2_ production (Fig. 5d), which would contribute to the pTau accumulation in neurons (Fig. 5f), in turn leading to the damage of synapses (Fig. 5g) or even neuronal death (Fig. 5h). Previous research disclosed that the neuroinflammatory response was also partly promoted by the inflammatory factors, such as TNFα and IL-1β, which released from reactive astrocytes^40^. On the contrary, we failed to detect any increase of cytokines released from co-cultured astrocytes/neurons under the exposure of AlNPs, suggesting that only a few inflammatory molecules were produced by AlNP-activated astrocytes. This further supported our speculation that H_2_O_2_, instead of inflammatory cytokines produced from reactive astrocytes, would be engaged in the pTau accumulation and neuronal impairment. In addition, AlNPs may cause neuronal death without the mediation of reactive astrocytes; thus, the direct impact of AlNPs directly on neurodegeneration should be studied in the future. Hence, our results clarified that exposure to AlNP poses a significant risk to the brain, increasing the likelihood of developing neurodegenerative disorders associated with pTau, one of key factors proceeding neurological disroders^15,33,41–44^.

In this study, we visualized the penetration of AlNPs to the brain due to the impairment of BBB, which triggered astrocyte activation, tauopathy, and neuronal loss, consequently (Supplementary Fig. 13). We believe that our study will provide profound and valuable insights into understanding the mechanisms of brain disorders occurred by ambient AlNP, alarming the severity of industrial air pollutants and urging utmost solution for workers in the industry area. Furthermore, we envision that our hNVUI model will provide a platform for high-throughput analyses for new drugs testing specifically targeting neurovascular diseases, which may foster the development of those incurable diseases.

## Methods

### Cell culture

Immortal human endothelial cells (h3MEC/D3 cells) were purchased from Cedarlane Laboratories (Ontario, Canada) and grown in the culture dish coated with 100 μg/mL collagen Type I-coated (Corning Inc., NY, USA) with EC proliferation medium (ECPM) consisting of endothelial cell growth basal medium-2 (EBM-2, Lonza, Basel, Switzerland), 1% v/v penicillin–streptomycin (Sigma-Aldrich, St. Louis, MO), 1.4 μM hydrocortisone, 10 mg/mL acid ascorbic, 1% v/v chemically defined lipid concentrate, 10 mM HEPES (Gibco-BRL, Gaithersburg, MD), 20 ng/mL bFGF (Stemgent, Cambridge, MA,USA), and 5% v/v fetal bovine serum (FBS, Sigma-Aldrich). After reaching 90% of cell confluency, we detached the cells with 2.5% Trypsin EDTA (Sigma-Aldrich), resuspended in fresh ECPM, and sub-cultured in new culture dish coated with 100 μg/mL collagen Type I.

Human neural progenitor cells (ReN cells, EMD Millipore, Billerica, MA, USA) were grown in the culture dish coated with 1% v/v Matrigel (Corning Inc.) with PM consisting of DMEM/F12 medium (ThermoFisher Scientific), 0.1% v/v Heparin (Stemcell Technologies), 2% v/v B27 serum free supplement (ThermoFisher Scientific), 1% v/v PSA antibiotic solution (Lonza), 20 μg/mL bFGF, and 500 ug/mL EGF (Sigma-Aldrich). Once the cells reached 80% confluency, ReN cells were detached using Accutase (Gibco-BRL), resuspended in fresh PM, and sub-cultured in new culture dish coated with 1% v/v Matrigel.

Immortalized human astrocytes (SV40 cells, ABM Inc., Richmond, BC, Canada) were grown in the culture dish coated with 40 μg/mL collagen Type I with astrocyte PM (ACPM) consisting of Prigrow IV (ABM Inc.), 10 ng/mL EGF, 1% v/v penicillin–streptomycin, 2 mM L-glutamine (Gibco-BRL), and 5% v/v FBS. The cells were detached with 2.5% Trypsin EDTA after reaching to the confluence of 80%, resuspended in ACPM, and sub-cultured in new culture dish coated with 40 μg/mL collagen Type I.

### Preparation of AlNP solution

Al_2_O_3_ nanoparticles (AlNPs) were purchased from PlasmaChem GmbH (Berlin, Germany). The average size and zeta potential of nanoparticles in the deionized water are 40 nm and 47.18 ± 2.829 mV (mean ± SD). AlNPs were initially prepared as a stock solution (1 mg/mL) dispersed in dimethyl sulfoxide (DMSO, Biosesang Inc., Seongnam-si, Korea). Before usage, we diluted in the proper culture media and sonicated for 30 min at room temperature (RT) to disperse the particles in the solution^15^. The final concentrations and the selection of culture media were varied depending on experimental settings and stated in the manuscript.

### Chip fabrication

To fabricate a mold of device, SU-8 (MicroChem, Newton, MA) was negatively patterned onto a 4-inch silicon wafer using the photolithography technique. Details related to the device fabrication were described in our previous study^45^. To prepare replica mold, we mixed Polydimethylsiloxane (PDMS) and curing reagent (Sylgard 184 A/B, Dowhitech, Goyang-si, Korea) at 10:1 ratio, poured the mixture onto the patterned silica wafer, and incubated the mold at 60 °C for 4 h for the solidification. The cured PDMS replica was peeled off from the mold, and holes were created for fluid reservoirs. Plastic chambers for medium reservoirs were fabricated with a computer-controlled Zing laser cutter (Epilog Laser, Golden, CO) with a 6-mm-thick acrylic plate. The replicated PDMS and plastic layers were glued together using PDMS. The resultant assembly was irreversibly bonded to a customized glass-bottomed uni-well plate (MatTek, Ashland, MA) by oxygen plasma treatment (Plasma Etch, Carson City, NV). In prior to the cell culture on the device, we coated each chamber with 1% v/v Matrigel diluted in DMEM/F12 for 1 h and washed it with Dulbecco’s phosphate-buffered saline (DPBS, Lonza, Hopkinton, MA) thoroughly.

### Preparation of hNVUI models

We developed the hNVUI model to investigate the underlying mechanisms of BBB-penetrating AlNPs promoting astrogliosis and neuronal damage. We coated both R.C. and L.C. of microfluidics with 10 μL of poly D-lysine (PDL, 1.0 mg/mL, Sigma-Aldrich), incubated at RT for 20 min, and washed with phosphate-buffered saline (PBS, Biosesang Inc.). The R.C. was further coated with 1% v/v Matrigel, incubated at 37 °C for 30 min, and washed with PBS thoroughly. Afterward, we loaded 10 μL of ReN cells (5 × 10^6^ cells/mL) to R.C. and incubated the devices in a 5% CO_2_ cell culture incubator at 37 °C for 30 min to ensure the completion of cell attachment to the surface. Afterwards, we added 50 μL of fresh differentiation media (DIM) and exchanged the one-half volume of DIM every 3.5 days until the progenitor cells were fully differentiated into neurons and astrocytes (approximately 3 weeks). The replacement of one half of medium every 3.5 days was designed to prevent any unexpected cell damage, caused by the shortage of nutrients in our 3D cultured models^15,32,38^. In prior to ECs loading, we coated L.C. with 2 mg/mL collagen Type I at 37 for 30 min and washed with PBS. We loaded 10 μL of ECs to collagen-coated L.C. at the density of 10^7^ cells mL and placed the device in a 5% CO_2_ cell culture incubator at 37 °C for 4 days. It should be noted that we tilt the device at 45° so that ECs could form BBB on the side wall of L.C. facing R.C. by gravity. Once the completion of BBB formation, we added 1 ng/mL of AlNPs to L.C. and incubated in a 5% CO_2_ cell culture incubator at 37 °C for 4 days.

### Other in vitro models

For the single-cultured BBB model, we detached h3MEC/D3 ECs with 2.5% Trypsin EDTA, resuspended in fresh ECPM, and loaded to 96 well-plates (ThermoFisher Scientific, Waltham, MA) coated with 100 μg/mL collagen Type I at the seeding density of 20,000 cells/well. To increase BBB tight-junctions, we performed the serum starvation by changing the culture media from ECPM to ECDIM for 4 days.

For the co-cultured model of neurons and astrocytes, ReN neural progenitor cells were detached using Accutase and loaded to 96 well-plates coated with 1% v/v Matrigel at the seeding density of 10,000 cells/well. ReN were cultured with DIM for 3 weeks to induce the differentiation of neural progenitor cells into neurons and astrocytes.

For the single-cultured astrocytes, SV40 astrocytes were detached with 2.5% Trypsin EDTA after reaching to the confluence of 80%, resuspended in ACPM, and seeded on 96 well-plates coated with 40 μg/mL collagen Type I with ACPM at the seeding density of 10,000 cells/well.

### Assessment of BBB permeability

To investigate the effects of AlNPs on the tightness of BBB, we assessed the permeability rate of 40 kDa FITC–dextran (Sigma-Aldrich) with or without treatment of AlNPs. Upon the completion of AlNP treatment, we added 10 μg/mL of 40 kDa FITC-dextran to L.C. of hNVUI and collected the conditioned medium from R.C. side every 6 h up to 24 h. We measured the concentration of BBB-penetrating FITC-dextran by Bio Tek Gen5 (Agilent Technologies, Santa Clara, CA, USA). The apparent permeability of each sample across the BBB (*P*_BBB_) was calculated by Eq. 1 as described previously^15,46^:

1 $$ {\text{P}}_{{{\text{BBB}}}} = \frac{{ C_{L} }}{{C_{0} }} \times \frac{V}{S \cdot t} $$

In this equation, C_L_ (μg/mL), C_0_ (μg/mL), V (cm^3^), S (cm^2^), and t (s) represent the concentration of FITC-dextran in R.C., the initial concentration of FITC-dextran in L.C., the volume of R.C., the surface area of microchannels connecting L.C. and R.C., and the time for BBB penetration, respectively.

### Assessment of ROS

We treated single-cultured astrocytes with 1 ng/mL of AlNPs for 2 days and co-cultured neurons and astrocytes with 10 pg/mL of AlNPs for 4 weeks. Afterward, we stained the models with 10 μM of CellROX™ green reagent (ThermoFisher Scientific), fluorescent probes detecting the intracellular ROS, for 30 min in a 5% CO_2_ cell culture incubator. We washed the models with PBS for three times and measured fluorescent intensity by a fluorescence microscope equipped with a FITC filter (Nikon TiE microscope, Nikon, Japan) in real-time. We investigated any fold changes in the fluorescent intensity representing the accumulated ROS in the cells by using NIS-Elements software (Nikon).

### Assessment of NO

We treated single-cultured ECs or astrocytes with or without 1 ng/mL of AlNPs for 2 days and assessed the level of NO released from ECs or astrocytes by using nitric oxide indicators, DAF-FM™ (ThermoFisher Scientific). Briefly, cells were washed with PBS and 10 μM of DAF-FM™ for 30 min in a 5% CO_2_ cell culture incubator. We rinsed the cells with PBS three times, added the culture medium, and incubated them for an additional 20 min to ensure the de-esterification of the diacetates in the cells. Finally, spontaneous production of NO was assessed in real-time by using a fluorescence microscope equipped with a FITC filter. We analyzed the fluorescent intensity representing NO by using a NIS-Elements software.

### Assessment of cellular senescence

We treated single-cultured ECs with or without 1 ng/mL of AlNPs for 2 days, fixed the cells with 4% paraformaldehyde (Biosesang Inc.) at RT for 15 min, and washed the cells with PBS supplemented with 0.1% v/v Tween ®20 (PBST) three times. Next, senescence detection solution assessing the activity of SA-β-gal was prepared by diluting CellEvent™ senescence green probe into prewarmed CellEvent™ senescence buffer (ThermoFisher Scientific) at the ratio of 1:1000, which was treated to cells immediately. The samples were prevented from light and incubated at 37 °C for 2 h without CO_2_. Afterwards, we discarded the detection solution, washed with PBS three times, and captured fluroescent images by using a fluorescence microscope equipped with a FITC filter. The fluorescent intensity indicating the activity of SA-β-gal was assessed by using a NIS-Elements software.

### Immunocytochemistry

Upon the completion of model preparation and AlNP treatment, we fixed the cells with 4% paraformaldehyde at RT for 15 min and washed with PBST three times. For the cell membrane permeabilization, cells were treated with PBST supplemented with 0.1% v/v Triton™-X 100 (Sigma-Aldrich) at RT for 15 min and washed with PBST three times. Next, cells were incubated with PBST supplemented with 2% v/v bovine serum albumin (BSA, Bovogen, Melbourne, VIC, Australia) at RT for 1 h for blocking. Cells were treated with primary antibodies diluted in the blocking solution at their appropriate working dilution ratios and incubated at 4 °C overnight. Cells were rinsed with PBST three times and incubated with secondary antibodies diluted in the blocking solution at 1:200 ratio along with 1% v/v Hoechst. The details of primary and secondary antibodies, including dilution ratios and other information, have been given in Table S1 (Supporting information). Afterwards, cells were washed with PBST five times and visualized by a fluorescence microscope. NIS-Elements software was used to assess the fluorescent intensity.

### Assessment of H_2_O_2_

The Amplex™ red hydrogen peroxide assay kit was purchased from ThermoFisher to detect the level of H_2_O_2_ in the conditioned medium. Samples were collected, mixed with H_2_O_2_ solution at the 1:1 ratio, and then incubated for 30 min at RT, protected from light. After incubation, the absorbance was measured using a microplate reader (Bio Tek Gen5).

### Statistical analysis

All the statistical data was performed as mean ± standard deviation using GraphPad Prism 9 software (Graphstats Technologies, San Diego, CA, USA). Briefly, unpaired two-tailed t-test was used to compare the statistical significance and *p* < 0.05 was assumed to be statistical significance. Symbols including ns, *, **, ***, **** were used to denote as no significance, *p* < 0.05, *p* < 0.01, *p* < 0.001, *p* < 0.0001 respectively.

### Fluorescence imaging quantification

Nikon TiE microscope was employed to take fluorescent images. To quantify the expression levels of representing markers after immunocytochemistry, Regions of Interest (ROIs) with uniformly sized area were selected randomly in each captured image, after which the average fluorescent intensity of the object ROIs was measured by NIS-Elements software. To assess the contact area (Fig. 1g), we selected the saturated region in the peripheral boundary of single cell from fluorescent images for VE-cad by Image J software. We also selected the body parts of cells from the same images and analyzed the size of ECs (Fig. 1h) by NIS-Elements software.

### Supplementary Information

Supplementary Information.

## Data Availability

All data have been included in the manuscript and the supplementary information.
