# Supplementary material for: Environmental aluminum oxide inducing neurodegeneration in human neurovascular unit with immunity
Source: Sci Rep. 2024 Jan 7;14:744. doi: 10.1038/s41598-024-51206-4 (PMC10772095; doi:10.1038/s41598-024-51206-4)
Supplement: Supplementary file 1 — Supplementary Information. [file 41598_2024_51206_MOESM1_ESM.docx]

**Environmental Aluminum Oxide Inducing Neurodegeneration in Human Neurovascular Unit with Immunity**

Yingqi Xue^1,2^, Minh Tran^1-3^, Yen N. Diep^1-3^, Seonghun Shin^4^, Jinkee Lee^1,4^, Hansang Cho^1-3^* and You Jung Kang^1,2^*

^1^Institute of Quantum Biophysics, Sungkyunkwan University, Suwon, Republic of Korea.

^2^Department of Biophysics, Sungkyunkwan University, Suwon, Republic of Korea

^3^Department of Intelligent Precision Healthcare Convergence, Sungkyunkwan University, Suwon, Republic of Korea

^4^School of Mechanical Engineering, Sungkyunkwan University, Suwon, Republic of Korea

*, co-corresponding author; E-mail: [h.cho@g.skku.edu](mailto:h.cho@g.skku.edu), youj.kang@gmail.com


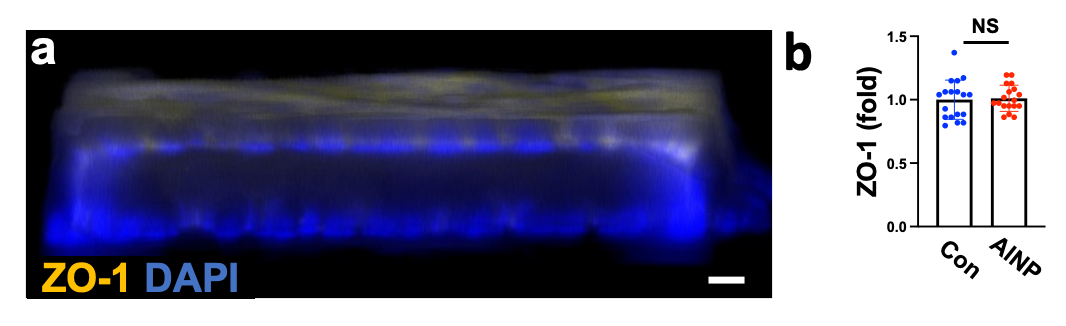


**Supplementary Figure 1.** **Expression level of ZO-1 in hNVUI model.** (a) Fluorescent image of Zonula Occluden-1 (ZO-1) and DAPI in the constructed 3D BBB model. (b) Quantitative results of ZO-1 level were represented as bar graphs (n=20). Scale bar, 30 µm. All data are presented as mean ± SD measured by two-tailed unpaired Student’s t-test. NS, no significance.


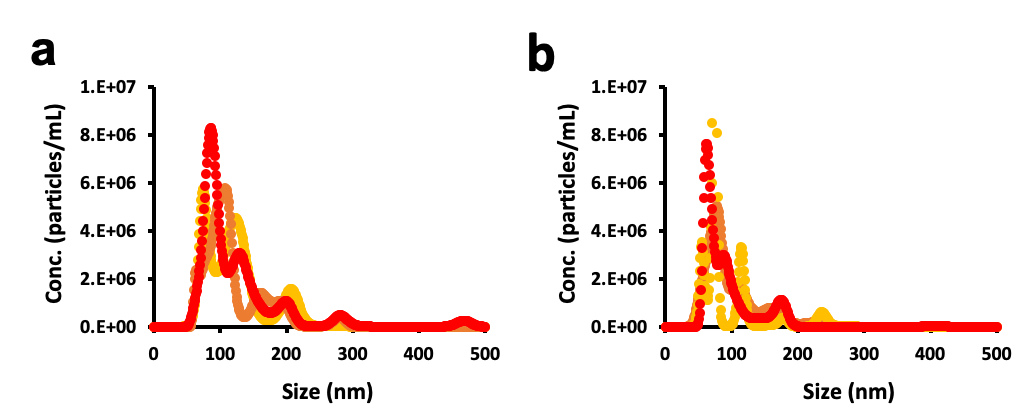


**Supplementary Figure 2. Penetration of AlNPs through BBB in hNVUI model.** To validate the penetration of AlNP across the BBB, we added 1 ng/mL of AlNPs to the blood compartment and took the samples from both blood and brain compartments after 4 days. We estimated the concentration of AlNPs a) before (2.95 X 10^8^ particles/mL) and b) after penetrating BBB (1.73 X 10^8^ particles/mL) by Nanoparticle Tracking Analysis (NTA) and found that approximately 14.6% of AlNPs penetrated BBB per a day. Each color represents a single measurement, repeated 3 times.


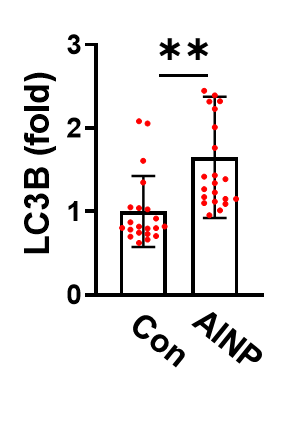


**Supplementary Figure 3. Phagocytic activity of astrocytes induced by AlNP.** Quantitative results of LC3b expressed in the astrocytes were assessed and represented as bar graphs (n=20). All data are presented as mean ± SD measured by two-tailed unpaired Student’s t-test. **, p< 0.001.


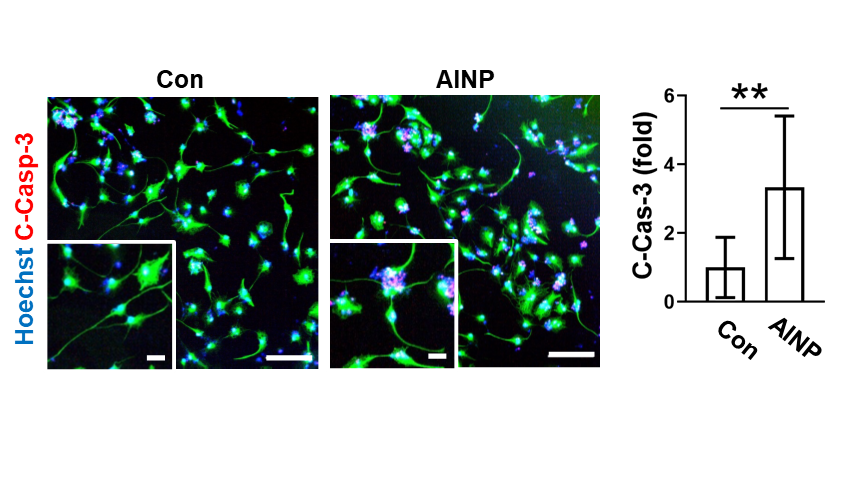


**Supplementary Figure 4. Induction of apoptotic cells by AlNP treatment.** Quantitative results of C-Cas-3 expressed in the co-cultured neurons and astrocytes were assessed and represented as bar graphs (n=20). All data are presented as mean ± SD measured by two-tailed unpaired Student’s t-test. **, p< 0.001.


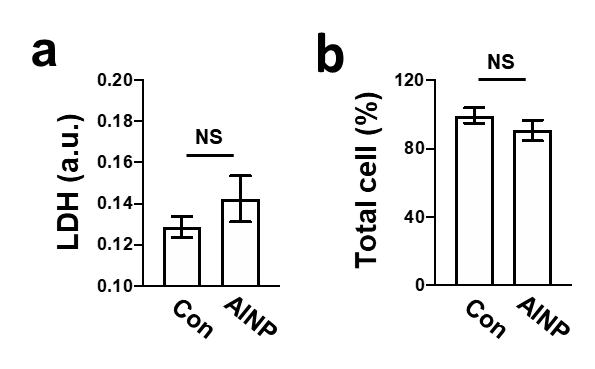


**Supplementary Figure 5. Impact of BBB-penetrated AlNP on the viability brain unit.** Quantification of a) the entire viability (n=5) and b) cell population (n=10) in the brain unit were represented as bar graphs. All data are presented as mean ± SD measured by two-tailed unpaired Student’s t-test. NS, no significance.


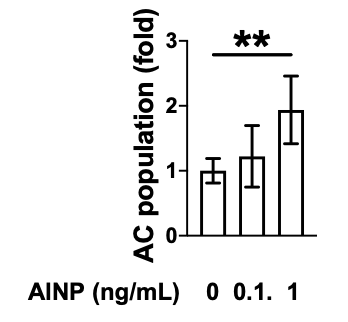


**Supplementary Figure 6. The number change of astrocytes (AC) with the treatment of AlNP.** The fold change of astrocyte population was represented as bar graphs (n=10). All data are presented as mean ± SD measured by two-tailed unpaired Student’s t-test. **, p< 0.01.


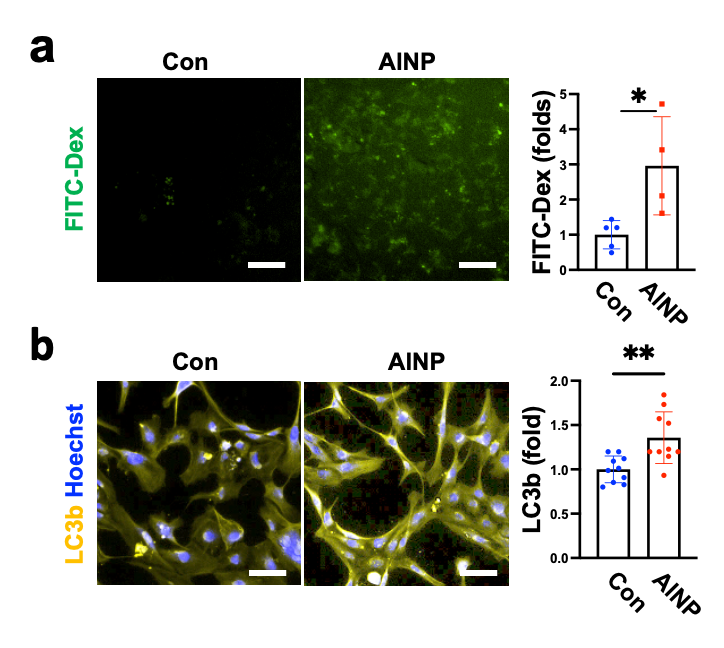


**Supplementary Figure 7. Assess the phagocytic function of astrocytes treated** **with AlNP.** To assess the phagocytosis activity of astrocytes, astrocytes were treated with 1 ng/mL AlNP for 2 days followed by 10 µM FITC-dextran (M.W. 40 kDa) for 2 hours. Quantitative results of a) FITC-dextran phagocytosed by astrocytes (n=5) and b) LC3b (n=10) were represented as bar graphs. Scale bars, 50 μm. All data are presented as mean ± SD measured by two-tailed unpaired Student’s t-test. *, p< 0.05 and **, p< 0.01.


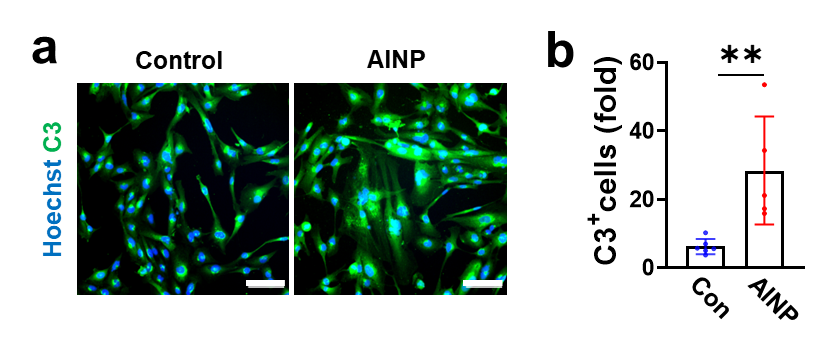


**Supplementary Figure 8. A1-like astrocytic reactivity induced by AlNP.** (a) Representative immunofluorescence images showing C3 in astrocytes. (b) Quantification of C3-positive cells was represented as bar graphs (n=6). Scale bars, 100 µm. All data are presented as mean ± SD measured by two-tailed unpaired Student’s t-test. **, p< 0.01.


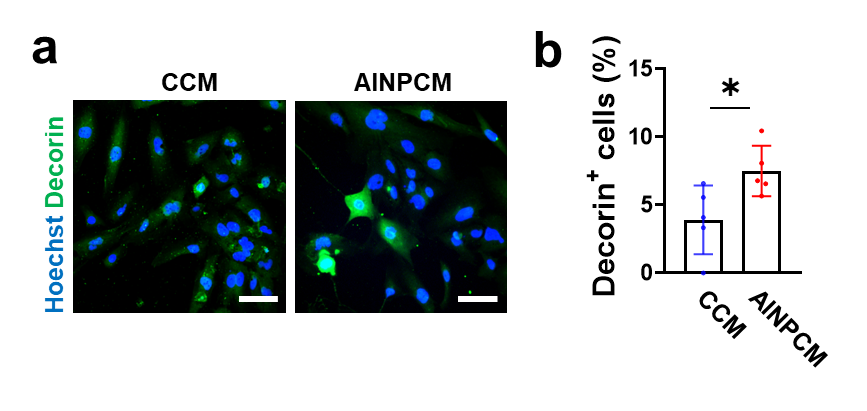


**Supplementary Figure 9. Influence of AlNP-treated ECs on the astrocytic reactivity.** (a) Representative immunofluorescence images showing Decorin in astrocytes with the treatment of CCM or AlNPCM. (b) Quantification of Decorin-positive cells was represented as bar graphs (n=6). Scale bars, 100 µm. All data are presented as mean ± SD measured by two-tailed unpaired Student’s t-test. *, p< 0.05.


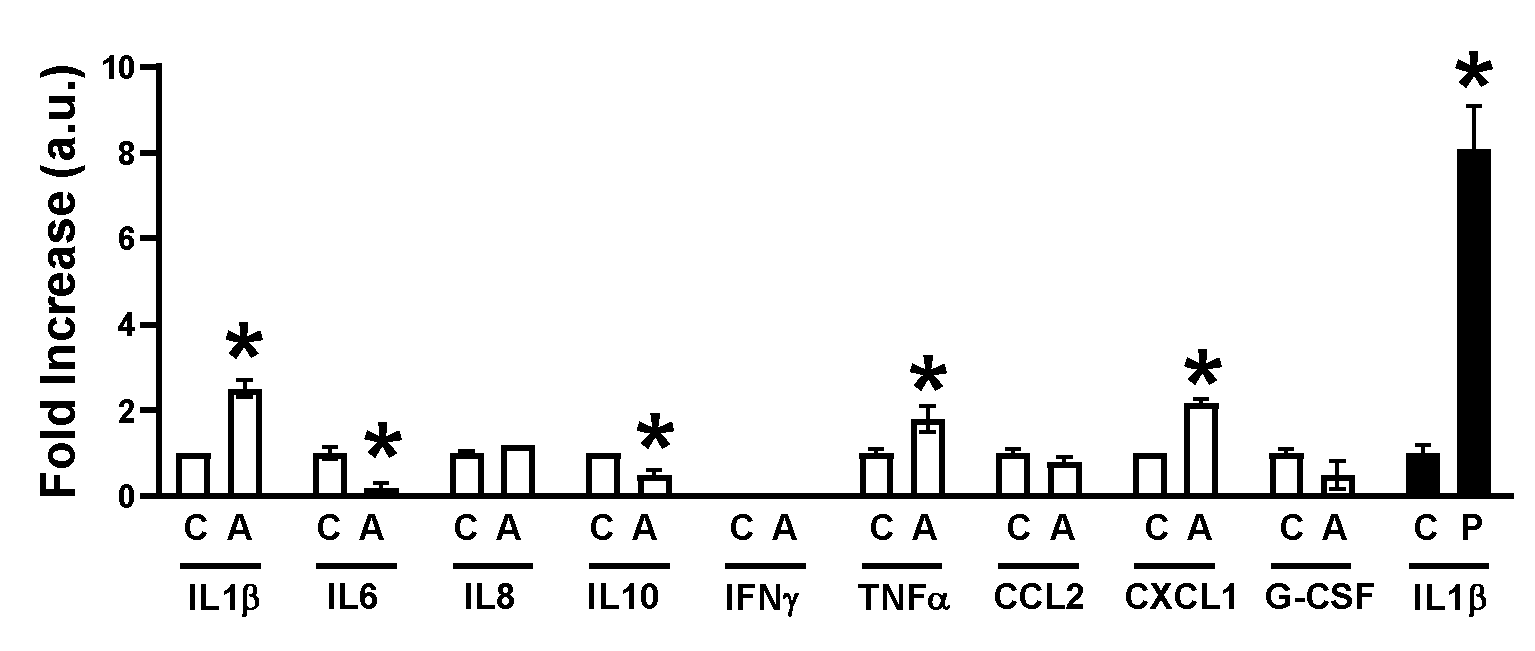


**Supplementary Figure 10. Assessment of cytokines in the conditioned media.** C: conditioned media from control ECs, A: conditioned media from AlNP-treated ECs, P: conditioned media from PM-treated ECs. Data represent means ± SD. *, p< 0.05.


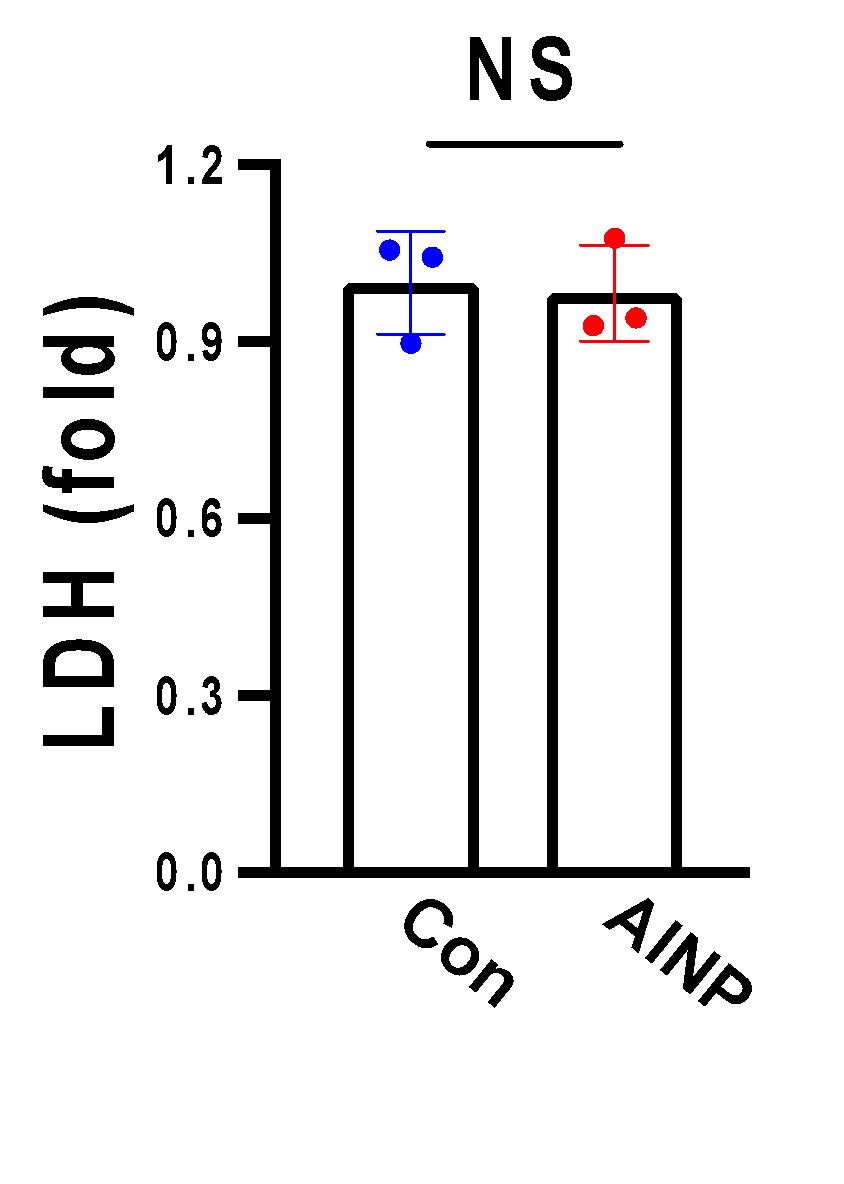


**Supplementary Figure 11.** **EC cytotoxicity with and without 1 ng/mL AlNP treatment for 4 days was examined.** The fold change of LDH released from ECs were represented as bar graphs (n=3). All data are presented as mean ± SD measured by two-tailed unpaired Student’s t-test. NS, no significance.


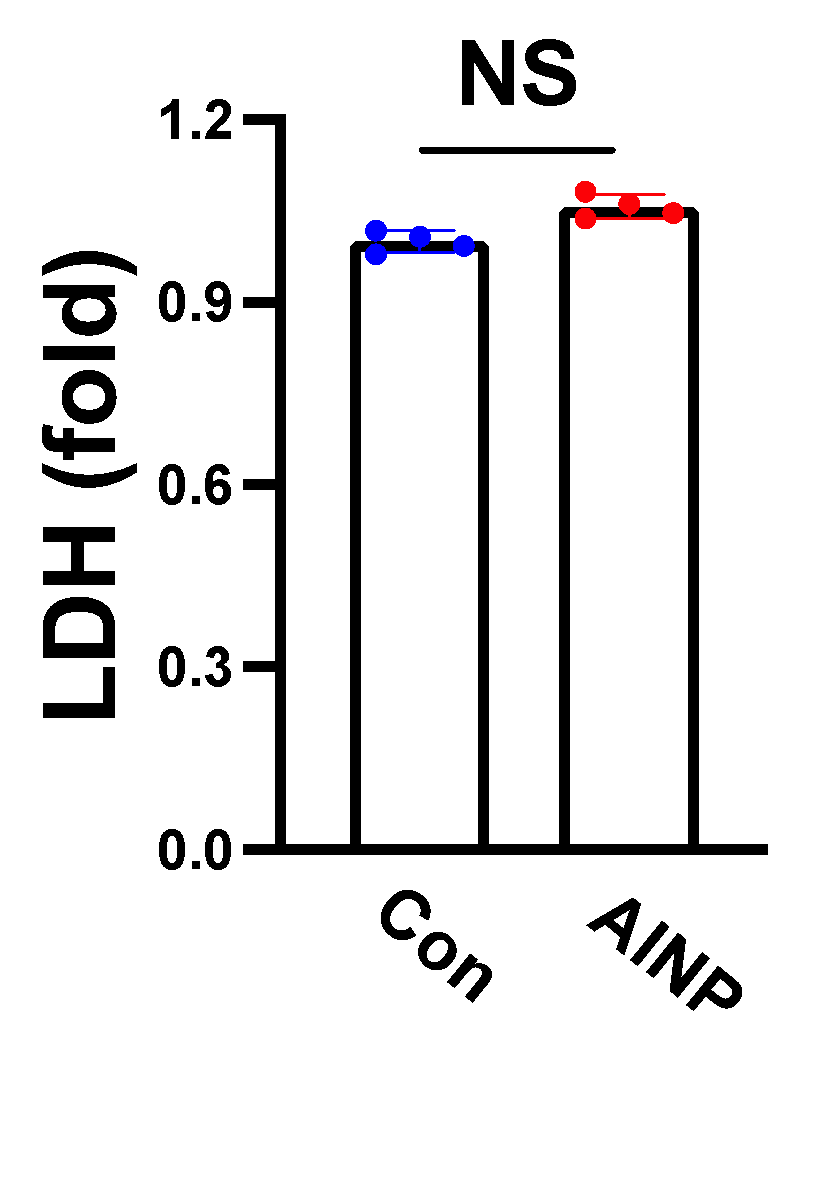


**Supplementary Figure 12.** **AC cytotoxicity with and without 1 ng/mL AlNP treatment for 4 days was examined.** The fold change of LDH released from ACs were represented as bar graphs (n=4). All data are presented as mean ± SD measured by two-tailed unpaired Student’s t-test. NS, no significance.


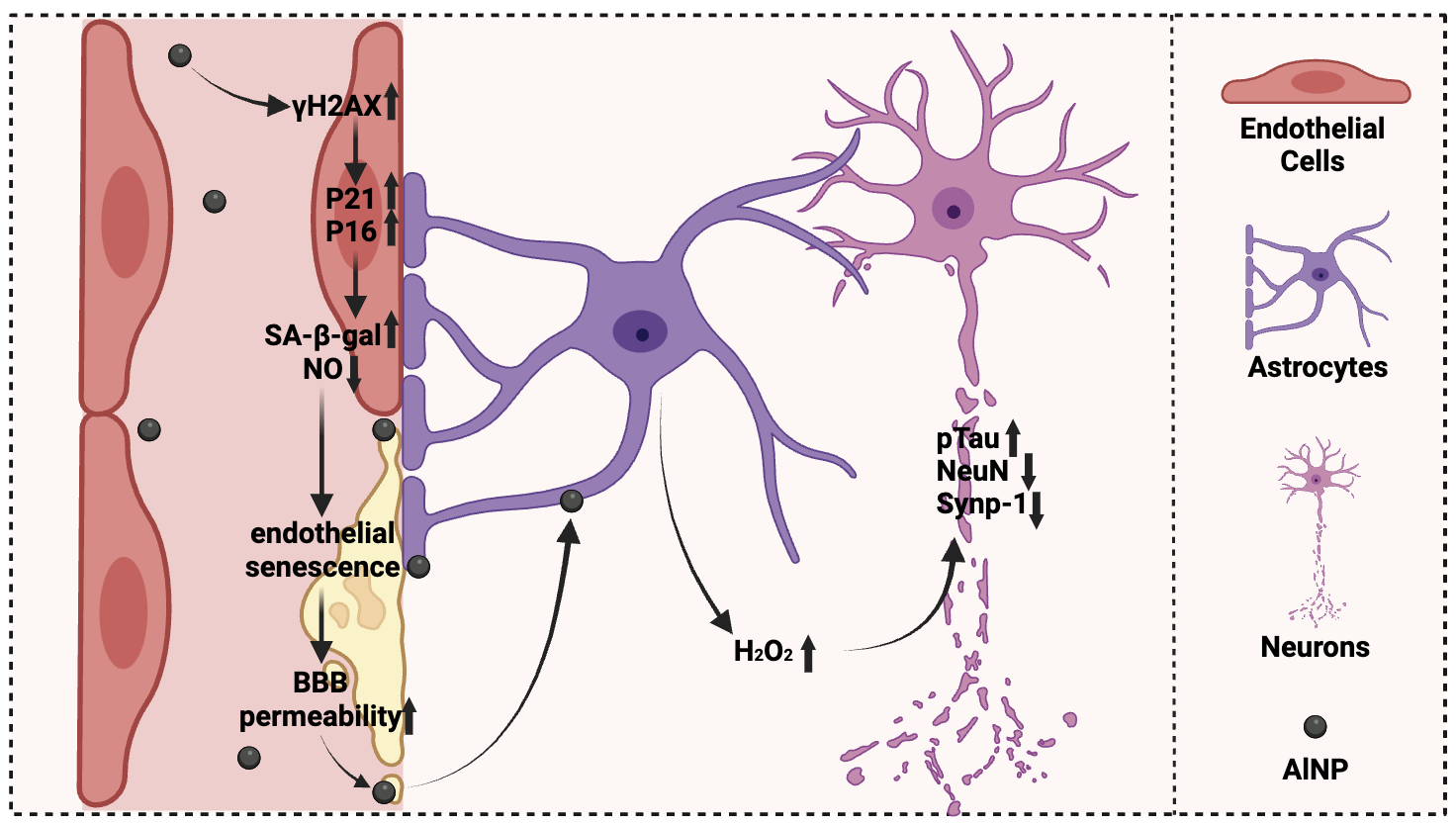


**Supplementary Figure 13.** **Summary of this study.** AlNPs triggered endothelial senescence by p21/p16 pathways, which elevated the BBB permeability. The BBB penetrating AlNPs then facilitated astrogliosis followed by neurodegeneration in the brain region.

**Table S1.** Antibodies used in the study

| **Antibodies** | **Company** | **Catalog#** | **Dilution ratio** |
| --- | --- | --- | --- |
| VE-cad | ThermoFisher Scientific | 36-1900 | 1:100 |
| GFAP | Sigma-Aldrich | AB5541 | 1:200 |
| Tuj-1 | NOVOS | NBP1-4256 | 1:200 |
| γH2AX | ThermoFisher Scientific | MA1-2022 | 1:400 |
| P21 | Abcam | AB227443 | 1:100 |
| P16 | Sigma-Aldrich | SAB56000308 | 1:100 |
| iNOS | ThermoFisher Scientific | PA1-036 | 1:200 |
| pNFkB | Cell Signaling | #3031S | 1:200 |
| ALDH1L1 | Abcam | AB190298 | 1:200 |
| Synapsin-1 | Abcam | AB254349 | 1:200 |
| pTau | ThermoFisher Scientific | MN1020 | 1:200 |
| Cleaved Caspase-3 | Cell Signaling | 9661S | 1:200 |
| Decorin | Abcam | ab175404 | 1:200 |
| C3 | Abcam | ab181147 | 1:200 |
| LC3b | Santa cruz Biotechnology | sc-376404 | 1:200 |
| Goat anti-mouse2’ ab  Alexa 594  Alexa 647 | ThermoFisher Scientific | A21216  A10524 | 1:200 |
| Goat anti-rabbit 2’ ab  Alexa 647 | ThermoFisher Scientific | A32733 | 1:200 |
| Goat anti-chicken2’ ab  Alexa 647 | EMD millipore | AP503H | 1:200 |
